# Photo Quiz: A 22-year-old leukemic patient with diarrhea

**DOI:** 10.1128/jcm.00312-24

**Published:** 2025-01-31

**Authors:** Debasish Biswal, Bijay Ranjan Mirdha

**Affiliations:** 1 Department of Microbiology, All India Institute of Medical Sciences 233643 , New Delhi, India; Mayo Clinic Minnesota, Rochester, Minnesota, USA

## Abstract

Read the full article for the answer.

## PHOTO QUIZ‍

**Fig 1:**
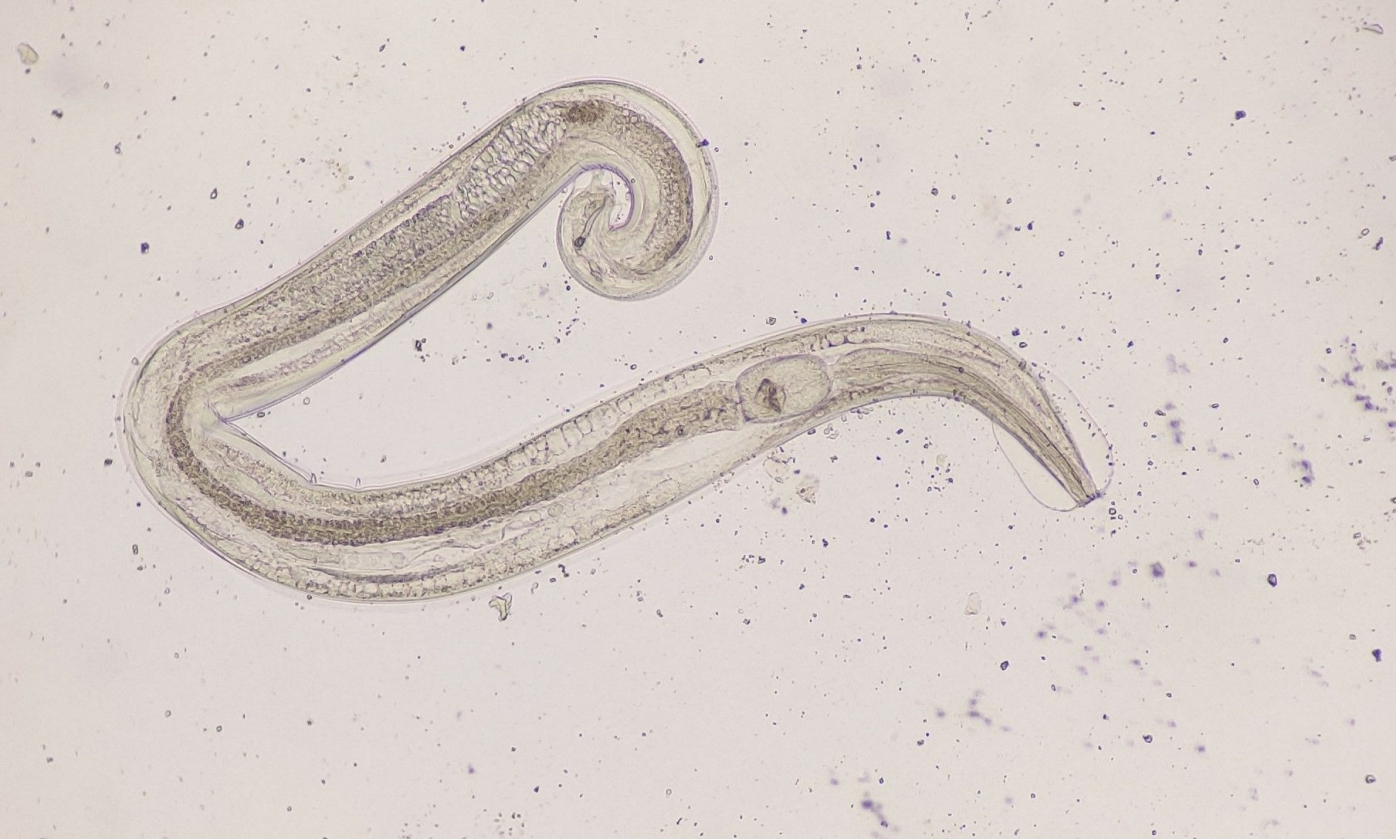
Adult worm detected on saline wet mount examination of the stool sample.

A 22-year-old male diagnosed with acute myeloid leukemia presented with intermittent low-grade fever (37.2°C–37.7°C) and non-mucoid diarrhea for the past 10 days. There was no history of vomiting. Diarrhea was not associated with abdominal pain and weight loss. No other significant history or any other known illnesses could be documented. On clinical examination, the abdomen was soft, not tender, with no distension. His heart rate was 116 bpm with normal blood pressure. His complete blood counts were within normal limits, with a total leukocyte count of 10,400/µL (reference range, 4,000–11,000/µL), a differential leukocyte count with 52% neutrophils (reference range, 40%–80%), 21% lymphocytes (reference range, 20%–40%), 6% monocytes (reference range, 2%–10%), 1% basophils (reference range, 0%–1%), with eosinophil of 15.4% (reference range, 1%–6%). Considering the underlying condition and complaint of diarrhea, a wet mount of stool specimen was prepared and examined. Examination detected a worm measuring 1.8 mm in length and 0.2 mm in breadth, which is shown in Fig. 1.

## ANSWER TO PHOTO QUIZ

The worm detected was transparent and nonmotile. The anterior end was round followed by a muscular double-bulb esophagus flanked by cervical alae. The posterior end had a tightly curved tail with a copulatory spicule. The worm was identified as an adult male of *Enterobius vermicularis*. The patient was treated with oral albendazole 400 mg (1). Diarrhea subsided, but a repeat dose of albendazole was administered after 2 weeks as young *Enterobius vermicularis* tend to be resistant to single therapy and can cause re-infection.

More than one billion people are thought to be infected with enterobiasis (1, 2) primarily affecting school-going children aged 4–11 years. Transmission occurs by ingestion of embryonated eggs or through external auto-infection due to onychophagia (habits of biting nails) and lack of personal hygiene (3). Eggs hatch in the intestine and the larvae migrate to the cecum and develop into adult worms. After fertilization, male worms usually die, and gravid females migrate to the large intestine and lay eggs around perianal skin (3, 4). Enterobiasis is diagnosed through the cellophane tape test or commercially available collection devices applied to the perianal area. The examination is usually done the next day morning before defecation for higher diagnostic of characteristic ova (1, 2). Eggs are rarely detected in stool and can only be seen in 4%–5% of cases, besides 40% of affected individuals are asymptomatic (5). The present finding is interesting because male worms of *Enterobius vermicularis* are rare, and their size is relatively smaller (reference range: length 2.5–5 mm; diameter [0.1–0.2 mm]). Mebendazole (100 mg) and albendazole (400 mg) are commonly used therapeutic agents. They are administered as a one-time dose followed by a repeat dose 2 weeks later. Pyrantel pamoate (11 mg up to a maximum of 1 g given two weeks apart) can be used but avoided in children below 2 years of age.
